# The magnetite-based receptors in the beak of birds and their role in avian navigation

**DOI:** 10.1007/s00359-012-0769-3

**Published:** 2012-10-31

**Authors:** R. Wiltschko, W. Wiltschko

**Affiliations:** FB Biowissenschaften, J.W.Goethe-Universität Frankfurt, Siesmayerstraße 70, 60054 Frankfurt a.M, Germany

**Keywords:** Magnetite-based receptors, Pulse treatment, Trigeminal nerve, ‘Fixed direction’ responses, Magnetic ‘map’ component

## Abstract

Iron-rich structures have been described in the beak of homing pigeons, chickens and several species of migratory birds and interpreted as magnetoreceptors. Here, we will briefly review findings associated with these receptors that throw light on their nature, their function and their role in avian navigation. Electrophysiological recordings from the ophthalmic nerve, behavioral studies and a ZENK-study indicate that the trigeminal system, the nerves innervating the beak, mediate information on magnetic changes, with the electrophysiological study suggesting that these are changes in intensity. Behavioral studies support the involvement of magnetite and the trigeminal system in magnetoreception, but clearly show that the inclination compass normally used by birds represents a separate system. However, if this compass is disrupted by certain light conditions, migrating birds show ‘fixed direction’ responses to the magnetic field, which originate in the receptors in the beak. Together, these findings point out that there are magnetite-based magnetoreceptors located in the upper beak close to the skin. Their natural function appears to be recording magnetic intensity and thus providing one component of the multi-factorial ‘navigational map’ of birds.

## Introduction

The literature on the avian magnetic compass, showing its functional properties, the underlying physical radical pair processes and its association with the visual system, has been discussed in several recent reviews (e.g., Ritz et al. 2010; Ritz 2011; W. Wiltschko et al. 2011). The other avian magnetoreception system, based on magnetite and associated with the trigeminal system, has received less attention and has not been summarized in a review so far. In the present paper, we will describe the respective findings, mostly behavioral that support the existence of magnetite-based magnetoreceptors in the beak of birds and also provide some insight in their structure, their function and their role in avian navigation.

## Biogenic magnetite and its possible role in magnetoreception

Lowenstam (1962) described magnetite, a form of Fe_3_O_4_ in the denticles of the radula of chitons (Mollusca: Polyplacophora), where they seem to be used for hardening the teeth. This was the first time that magnetic material was found in living organisms and proved in principle that magnetic material can be produced by biological means. In the mid-1970s, Blakemore (1975) found intra-cellular magnetite crystals in bacteria, a discovery that was soon followed by many other reports describing the occurrence of magnetite in other bacterial species, protozoa etc., and also in higher animals (see Kirschvink et al. 1985a for a summary up to that date).

Bacteria use their magnetite inclusions for orientation: they become ‘north-seeking’ or ‘south-seeking’; when they are stirred up, they move along the magnetic field lines to reach the ground. This is a mere passive orientation, mediated by the force of the magnetic field acting upon the magnetite crystals. Yet it inspired Yorke (1979) to suggest that magnetite could also be part of magnetoreceptors in higher animals.

The magnetic properties of magnetite depend on the size of the particles: larger particles are multi domain with no net magnetization; smaller crystals are single domains (SD) with a stable magnetic moment, and even smaller ones are superparamagnetic (SPM)—they do not have a stable magnetic moment, but will align their magnetization by an external magnetic field. Kirschvink and Gould (1981) described several ways how magnetite particles could work in a receptor, discussing systems based on single domain magnetite, on superparamagnetic crystals or a combination of both. These first considerations on the function of magnetite in receptors were followed by others proposing modified models and considering various theoretical possibilities how magnetite-based receptors might work and what type of information they might convey (e.g., Kirschvink and Walker 1985; Shcherbakov and Winklhofer 1999; Davila et al. 2003; Fleissner et al. 2007; Solovyov and Greiner 2007; Walker 2008; Winklhofer and Kirschvink 2010).

## Magnetite found in birds

Birds were a group of special interest, because their ability to use information from the geomagnetic field for orientation had been demonstrated: migratory birds had been shown to use the magnetic field as a compass (e.g., W. Wiltschko 1968; Keeton 1971), and the behavior of pigeons in a magnetic anomaly suggested a possible use of magnetic components in the navigational ‘map’ (Walcott 1978).

The first report of magnetite in birds was published in 1979, when Walcott et al. (1979), measuring the remanence with a SQUID magnetometer, found permanently magnetic material, presumably single domain magnetite, in the head of pigeons between the brain and the skull, that is, at a location where a sensory function does not seem very likely. Other iron-rich structures were found in the nasal region of birds: based on histological studies with Prussian blue staining, Beason and Nichols (1984) and Beason and Brennan (1986) described such structures in Bobolinks (*Dolichonyx oryzivorus*: Emberizidae), a passerine migrant, and Williams and Wild (2001) reported similar structures in pigeons. The size of the structures and remanence measurements suggested single domain magnetite. Being associated with the ophthalmic branch of the trigeminal nerve, these structures were discussed as possible magnetoreceptors.

Another type of putative magnetoreceptors involving superparamagnetic magnetite was described in the skin of the upper beak of pigeons. Hanzlik et al. (2000) identified the iron-rich particles crystallographically as magnetite; histological and electron microscopic studies revealed the fine structure, indicating clusters of nanocrystals adjacent to or within dentrites of the ophthalmic nerves (Winklhofer et al. 2001; Fleissner et al. 2003, 2007). An independent histological study and remanence measurements by Tian et al. (2007) supported these findings. Falkenberg et al. (2010) reported similar structures in the beak of domestic chickens (*Gallus gallus*) and two species of migrating passerines, the Garden Warbler (*Sylvia borin*) and the European Robin (*Erithacus rubecula*: Turdidae). These findings have recently been challenged by Treiber et al. (2012) who claim that the iron-containing structures described as potential magnetoreceptors were merely macrophages.

Magnetic material, presumably magnetite, has also been described in other vertebrates. Cells containing single domain magnetite were also found in the nasal regions of fish from the genus *Oncorhynchus* (Salmonidae), and interpreted to play a role in magnetoreception (e.g., Kirschvink et al. 1985b; Mann et al. 1988; Walker et al. 1997; Diebel et al. 2000; Eder et al. 2012). Remanence measurements indicated magnetite in the newts (Brassart et al. 1999) and in the heads of bats (Tian et al. 2010). Ferrous inclusions, discussed to be magnetite, were also found the cornea of mole-rat (genus *Fukomys*, Bathyergidae) (Wegner et al. 2006).

## The pioneering studies by Beason and Semm

**Fig. 1:**
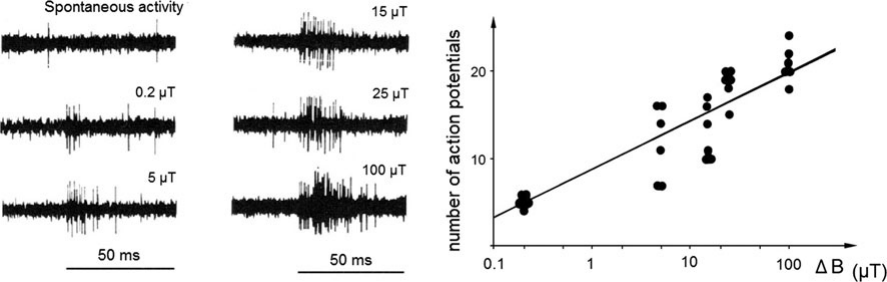
Electrophysiological recordings from the trigeminal ganglion of the bobolink. *Left* spontaneous activity and responses to various changes in the intensity of the vertical component of the magnetic field. The *lines * indicate the 50 ms of stimulation (from Semm and Beason 1990). *Right* recorded activity changes as a logarithmic function of the increased intensity (from Beason and Semm 1991)

Beason and Semm (1987, 1991); Semm and Beason (1990) recorded electrophysiological responses to magnetic stimulation from the ophthalmic nerve and the trigeminal ganglion of Bobolinks. Their stimuli consisted of changes in direction and intensity of the magnetic field produced by a set of coils. Figure 1a showed the responses to increases of the vertical components of the magnetic field; in Fig. 1b, the number of spikes is plotted as a function of the magnetic intensity, revealing a logarithmic relationship.

## From their findings, Beason and Semm drew two conclusions

1. The ophthalmic nerve mediates magnetic information,
2. This information is probably the information on magnetic intensity involved in the navigational ‘map’.

The first conclusion, namely that a branch of the trigeminal nerve mediates magnetic information, has been confirmed in a behavioral study with caged Bobolinks by the authors themselves (Beason and Semm 1996): injecting an anesthetic in the ophthalmic nerve suppressed the effect of a short, strong magnetic pulse (see Fig. 3a below). A conditioning study by Mora et al. (2004) produced corresponding results: after locally anesthetizing or sectioning the ophthalmic nerve, homing pigeons trained to respond to a small, strong magnetic anomaly could no longer perform the task. Similar results were obtained by Freire et al. (2012) with young Pekin Ducks (*Anas platyrhynchos* *domestica*) trained to associate a small, strong magnetic anomaly with food: here, too, anesthesia or sectioning the ophthalmic nerve led to a performance at chance level. A recent ZENK-study with European robins (Heyers et al. 2010) likewise confirms the involvement of the trigeminal system in magnetoreception: after magnetic stimulation, a number of ZENK-positive neurons were found in two areas in the trigeminal brain stem, and this number deceased significantly when the ophthalmic nerve was sectioned.

In all studies mentioned above, the magnetic stimuli used do not allow to decide whether the information mediated by the ophthalmic nerve was indeed information on intensity. Behavioral data that will be discussed below support this view, but recent findings show that the situation is rather complex.

## Pulse experiments to indicate magnetite

The electrophysiological responses recorded in the ophthalmic nerve after magnetic stimulation suggested these responses originate in the iron-rich structures found by the histological studies and the remanence measurements. Yet more direct evidence for an involvement of magnetite in orientation behavior seemed desirable. A diagnostic tool to identify magnetite is the response to a strong, short magnetic pulse, e.g., 0.5 Tesla with a duration of <5 ms: the pulse must be strong enough to remagnetize single domains, and it must be brief enough to prevent the magnetite crystals to rotate and align in the direction of the pulse. Since the orientation of the magnetite particles was unclear, it was to be expected that such a pulse would remagnetize roughly half of the single domains. A pulse would also disrupt clusters and chains of superparamagnetic magnetite, as experiments with model systems of ferrofluids showed (Davila et al. 2005). In both cases, receptors based on magnetite would be severely disrupted, and their input would be markedly changed. Other types of magnetoreception, like the radical-pair processes in the eye (Ritz et al. 2000, 2004), are not affected by the pulse, however.

**Fig. 2:**
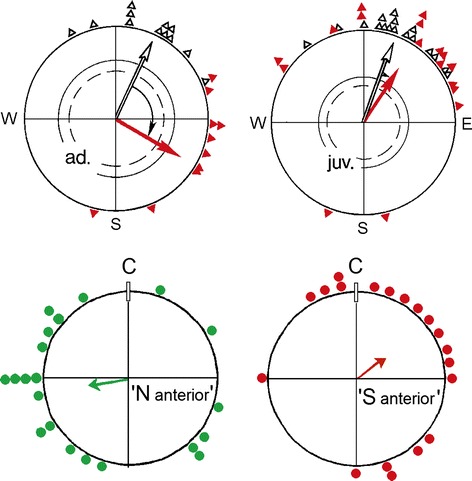
Orientation of migrants after a short, strong magnetic pulse. *Upper diagrams* responses of Australian Silvereyes showing that adult (*ad.*) birds respond with a marked deflection to the east, whereas juveniles (*juv.*) are unaffected. *Black open symbols*: behavior before pulsing; *solid red symbols*: behavior on the day of pulsing and the following day (data from Munro et al. 1997). *Lower* *diagrams* responses of bobolinks to pulses applied in two different directions. The headings are presented in relation to the orientation before the pulse (C) projected upward (data from Beason et al. 1995). The *arrows* represent the mean vector in relation to the radius of the circle = 1; the two *inner circles* are the 5 % (*dashed*) and 1 % significance border of the Rayleigh test

**Fig. 3:**
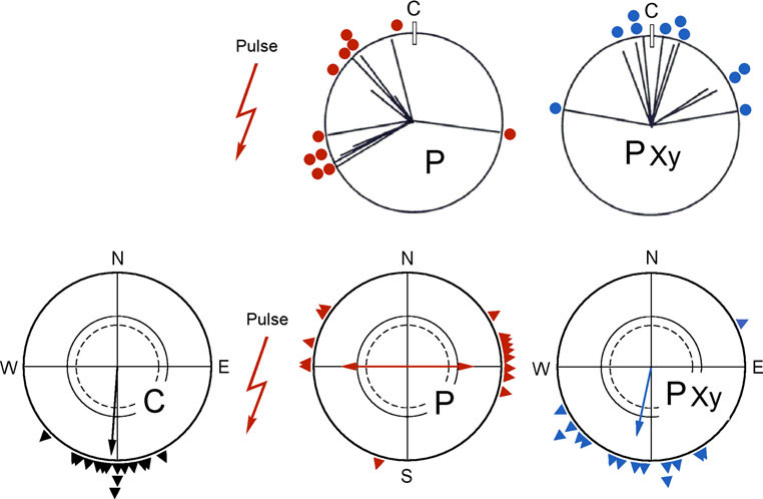
The response to the pulse can be abolished by preventing the information from the magnetite-based receptors reaching the brain—in that case, the birds head in their migratory direction with their magnetic compass, indicating that the pulse does not affect the compass. *Upper diagrams* response of Bobolinks to a pulse ‘south anterior (P) and when the ophthalmic branch of the trigeminal nerve was anesthetized with a local anesthetic (data from Beason and Semm 1996). *Lower diagrams* orientation of Australian Silvereyes before pulsing (C, *black symbols*) and after being subjected to the pulse without further treatment (P, *red symbols*) and with the upper beak locally anesthetized by a local anesthetic, externally applied (P Xy, *blue symbols*) (data from W. Wiltschko et al. 2009). Symbols as in Fig. 2

The first birds subjected to such a magnetic pulse were Australian Silvereyes (*Zosterops lateralis*), Australian migrants. In cage studies, the pulse was applied ‘south-anterior’, with the direction of the pulse being towards the beak in birds facing east in the geomagnetic field. This caused a roughly 90° shift to the east during spring as well as during autumn migration (Fig. 2a)—the effect was obviously independent from the migratory direction (W. Wiltschko et al. 1994, 1998). Interestingly, the effect of pulsing was only observed in old, experienced migrants; young birds that had been captured immediately after fledging did not respond to the pulse (Fig. 2b; Munro et al. 1997). Treating the ophthalmic nerve or upper beak of birds with the local anesthetic suppressed this pulse effect (Fig. 3; Beason and Semm 1987; W. Wiltschko et al. 2009).^1^

Beason et al. (1995) pulsed Bobolinks in two different directions, recorded their activity in cages and found that they deviated in different directions: the bird pulsed ‘south anterior’ headed to the right, those pulsed ‘north anterior’ to the left of their directions before pulsing (Fig. 2c). Deflections to different sides were also observed in homing pigeons that had been displaced after being pulsed ‘south anterior’ and ‘south left’ (Beason et al. 1997). Here, the observed effect was much smaller, probably because the pigeons were released and had, in contrast to the migrants tested in cages, other navigational factors available.

The pulse was found to alter the behavior of migrants only temporarily. The deviations described above are observed only on the day of pulsing and the two following days; after that the birds in the cage studies were disoriented, and about 8–10 days after the pulse, their orientation was back to normal—they again preferred their seasonally appropriate migratory direction (W. Wiltschko et al. 1994, 1998). This transient nature of the effect proved important when wild migrants were subjected to a pulse before they were released and their starting routes were tracked. A first study in America involving Catbirds (*Dumetella carolinensis*: Mimidae) proved inconclusive because the birds lingered in the area and took off only with considerable delay (Holland et al. 2009). In a second study in spring with European Robins and Reed Warblers (*Acrocephalus scirpaceus*: Sylviidae), many birds departed in the first days after pulsing, and here, the treated birds showed a significant deviation from the untreated controls, with the amount and direction of this deviation depending on the direction of pulsing (Holland 2010). A subsequent study with robins in autumn showed that juvenile birds were not affected by the pulse and those adult birds that took off within the first 10 days showed a significant deviation from their migratory direction, in contrast to those that departed later (Holland and Helm 2012).

Together, these findings provide some information on the receptors in question: the effect of the strong, short magnetic pulse on the orientation of birds indicates that the receptors are indeed based on magnetic material like magnetite. The observation that the deviations induced by pulsing depended on the direction in which the pulse is applied points out that the pulse does not silence the magnetite-based receptors altogether and that it just alters the information they mediate. At the same time, this observation also suggests that the magnetite particles in the receptors are not evenly or circular-symmetrically arranged, but extend differently to the various sides. From the behavioral point of view, it is interesting that only experienced, but not juvenile birds are affected, as this suggests that the mechanism involved is not innate, but based on experience.

## Single domains or superparamagnetic particles?

Pulse experiments were first proposed to find out whether the receptors controlling the respective behaviors contained magnetic material like magnetite. The observed effects of pulsing indeed indicate magnetite-based receptors, but it is not clear what type of particles is involved. Any interpretation of behavioral data in view of this question must necessarily remain speculative. Yet some findings give at least some hints.

The observation that the pulse effect is rather short-lived speaks against an involvement of single domain particles, because the new magnetization should be just as stable as the original one. Restoring the remagnetized particles seems rather unlikely, for this would mean that these particles would all have to be replaced within 10 days, the more, since the correct direction of magnetization could be difficult to determine under these circumstances. Recalibration of the altered input could have been possible for free-flying migrants, but seems unlikely for caged birds, because it is hard to see how they could have obtained the necessary information and new experience. The input from the damaged receptors could be simply ignored after a while, but the finding that a second, identical pulse affected the orientation again speaks against this possibility (W. Wiltschko et al. 2007). The second pulse, administered on day 16 after the first, led to a brief period of disorientation that lasted only 1 or 2 days; afterwards the Silvereyes again preferred their normal migratory direction. Rearrangement of disrupted clusters of superparamagnetic crystals, on the other hand, could roughly fall into the observed time frame (see Davila et al. 2005). The very short duration of the effect of the second pulse could be interpreted in the sense that repairing mechanisms had been activated and could be reactivated faster than after the first pulse.

Another approach involved pulsing the birds while they are exposed to a strong biasing field that aligns single domain particles (if sufficiently mobile) in one direction—in this case, a pulse parallel to the biasing field should have no effect, whereas an antiparallel pulse should remagnetize a maximum of particles, and thus lead to a marked change in orientation. The results of the two such experiments do not agree: Australian Silvereyes, pulsed parallel in a 100 μT biasing field showed the same axial deviations from their migratory direction as those pulsed antiparallel (W. Wiltschko et al. 2002), whereas Reed Warblers pulsed in a 320 μT biasing field seemed unaffected by a parallel pulse and showed an axial deviation when pulsed antiparallel (Holland 2010). The data of the pulse experiments available so far do not yet allow a decision on the type of particles involved in the magnetite-based receptors.

## What kind of information is mediated?

Another question concerns the type of magnetic information these receptors provide—directional information for compass use or a component of the navigational ‘map’. Theoretically, magnetite-based receptors could provide information on both, direction and intensity (e.g., Kirschvink and Gould 1981; Solovyov and Greiner 2007). But these are two different qualities of the magnetic field—we humans measure them with different instruments, a compass and a magnetometer. Hence, it would not be surprising if birds had specialized their magnetic sense in a similar way.

The electrophysiological study by Semm and Beason (1990) pointed towards magnetic intensity, and the observations of Munro et al. (1997) that young Australian Silvereyes remain unaffected by the pulse also speaks in favor of a learned system, which suggests the magnetite-based receptors provide a component of the navigational ‘map’ (see also Deutschlander et al. 2012). At the same time, the data of the respective study (Fig. 2b) clearly show that the young birds, despite the pulse treatment that disrupted their magnetite-based receptors, continued in migratory direction: obviously, their magnetic compass mechanism was not affected. This conclusion is also supported by findings that pulse-treated birds preferred their normal migratory direction when the altered input from the magnetite-based receptors was disrupted, either by anesthetizing the ophthalmic nerve (Fig. 3a; Beason and Semm 1996) or by anesthetizing the skin of the upper beak with the local anesthetic Xylocain, applied externally (Fig. 3b; W. Wiltschko et al. 2009). Testing pulse-treated Silvereyes in a magnetic field with the vertical component reversed showed that these birds located their changed course with their normal inclination compass (W. Wiltschko et al. 2006). Furthermore, in otherwise untreated Robins and Silvereyes, local anesthesia of the upper beak—the same treatment that disrupts the pulse effect—did not affect migratory orientation (R. Wiltschko et al. 2007, 2008).^2^ Zapka et al. (2009) obtained similar results by cutting the ophthalmic nerve.

All these findings clearly show that the magnetite-based receptors are *not* involved in the avian magnetic compass. The inclination compass represents a different mechanism based on different physical reactions, namely on radical-pair processes in the eye (Ritz et al. 2000, 2004). The magnetite-based receptors in the beak, in contrast, seem to contribute to the processes that determine the course to be pursued, the avian ‘map’ mechanism. In view of this, it appears most likely that the iron-rich structures mediate information on magnetic intensity.

In rainbow trouts, *Oncorhynchus mykiss,* two findings that might be parallel cases have been reported. In electrophysiological studies from a branch of the trigeminal nerve, Walker et al. (1997) found responses to changes in intensity, but not to changes in direction alone. In a study by Hellinger and Hoffmann (2012), fish were conditioned to magnetic stimuli; inactivation of the ophthalmic branch of the trigeminal nerve by a local anesthetic abolished the response to changes in intensity, but did not affect the response to changes in direction.

## ‘Fixed direction’ responses

The cage experiments with migratory birds reported so far were performed under ‘white’ or under very low intensity monochromatic lights from the short-wavelength end of the spectrum—here, the birds prefer their natural migratory direction. In total darkness, under red light or when short-wavelength light is combined with yellow light, birds change their behavior and head in directions that are different from their migratory direction—since these preferences do not show the seasonal change between autumn and spring, they are characterized as ‘fixed direction’ responses (see R. Wiltschko et al. 2010a). In contrast to migratory orientation by the inclination compass which ignores the polarity of the magnetic field (W. Wiltschko and Wiltschko 1972), these ‘fixed direction’ responses are polar responses, that is, they orient by the polarity of the magnetic field. This indicates that they must be based on a mechanism that is fundamentally different from the radical pair processes of the inclination compass.

**Fig. 4:**
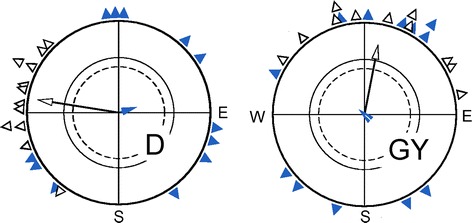
‘Fixed direction’ responses in Robins, observed under certain light regimes, can be disrupted by local anesthesia of the upper beak. D, orientation in total darkness; GY, orientation under a combination of monochromatic *green* and *yellow* light. *Black open symbols*: untreated controls; *blue solid symbols*: birds with the upper beak locally anesthetized with an externally applied local anesthetic (data from Stapput et al. 2008; R. Wiltschko et al. 2012). Symbols as in Fig. 2

Several such ‘fixed direction’ responses have been described in European Robins and Australian Silvereyes (e.g., R. Wiltschko et al. 2007, 2008, 2012; Stapput et al. 2008; for review, see R. Wiltschko et al. 2010a). The ones analyzed in detail so far could all be disrupted by local anesthesia of the upper beak (for examples, see Fig. 4)—the magnetic information involved appears to originate in the magnetite-based receptors located there. Obviously, under some extreme light conditions, the normal inclination compass is disrupted, and the receptors in the upper beak can take over and control the directional behavior of birds.

This means that the magnetite-based receptors do not only provide information on magnetic intensity, but under certain conditions also produce directional information. However, for reasons unknown, this information is not helpful for the birds—they cannot use it for locating their seasonally changing migratory direction. A biological significance of these ‘fixed direction’ responses to the magnetic field is not known; they have been discussed as possible relicts of an ancient, magnetite-based compass mechanism that has been replaced by the radical pair mechanism in birds (e.g., R. Wiltschko et al. 2010a), thus giving the magnetite-based receptors a chance to specialize for a different function. The ‘fixed directions’ depend on the respective light regimes and differ under the various colors and color combinations of light (see Fig. 4). Since the receptors in the upper beak cannot be directly affected by light, this suggests connections between the trigeminal and the visual system at higher levels in the brain.

## What is the natural function of the magnetite-based receptors?

So far, the behavioral evidence that indicates the occurrence of magnetite-based receptors in the upper beak involves highly unnatural stimuli: neither spatially limited strong anomalies, nor magnetic pulses or extreme light regimes exist in nature. This leads to the question about the natural role of these receptors. Some of the findings—the electrophysiological recordings by Semm and Beason (1990), the observation that young, inexperienced migrants are not affected by the magnetic pulse (Munro et al. 1997) and that the magnetic compass is not involved (Beason and Semm 1996; W. Wiltschko et al. 2006; R. Wiltschko et al. 2007; Zapka et al. 2009)—suggest that these receptors normally mediate magnetic information as a component of the navigational ‘map’. Pigeons have been shown to respond to temporal fluctuations of the geomagnetic field as reflected by the K-indices or the *A*_p_ index, with slight shifts in their initial orientation and a lower steadiness of their homing flight (Keeton et al. 1974; Schiffner and Wiltschko 2011), which suggests a sensitivity in the range of at least 20 nT. In Central Europe, the magnetic reference field increases by 2.5 nT/km towards 15° NNE; hence magnetic gradients could be helpful for navigation, provided that the spatial distribution of the geomagnetic field is fairly regular and that the birds are familiar with the local and regional magnetic topography.

**Fig. 5:**
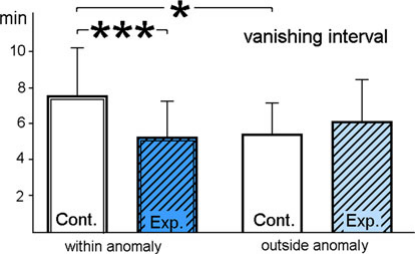
Vanishing intervals of untreated pigeons (*Cont.*, controls) and pigeons with the upper beak anesthetized by an externally applied local anesthetic (*Exp.*, experimentals) within an strong, irregular anomaly and outside in magnetically quiet terrain. The *columns* indicate mean and standard deviations. *Asterisks* indicate significant differences: *p < 0.05; ***p < 0.001 (data from R. Wiltschko et al. 2010a, b)

In the 1970s, Walcott (1978) released pigeons in a strong, irregular magnetic anomaly in the Northeastern USA and found that the quality of their orientation, represented by the vector length, decreased as the differences in intensity over a distance of 1 km increased. Yet at that time, nothing was known about the receptors that mediate the respective magnetic information. Walcott’s findings have recently been repeated in Germany with pigeons released in the Vogelsberg anomaly (R. Wiltschko et al. 2009); additionally, a marked effect on the vanishing interval (i.e., the time pigeons need to fly out of sight of observers with good binoculars, about 2.5 km) was observed. In a subsequent study, pigeons were released within the anomaly and outside in magnetically quiet terrain with the receptors in the upper beak temporarily deactivated by an externally applied local anesthetic. This affected their behavior considerably. Within the anomaly, the effect was somewhat beneficial: the pigeons with their beak anesthetized had longer vectors and were significantly faster to leave the site than the untreated controls. Anesthesia of the upper beak had obviously removed an impeding input. The controls were rather slow, significantly slower than when released outside the anomaly (Fig. 5; R. Wiltschko et al. 2010b). The hesitance of control pigeons to leave within the anomaly seemed to reflect confusion caused by the irregular magnetic conditions they encountered when flying around. These birds needed some time to realize that the magnetic field was not reliable and finally turn to other, non-magnetic cues. The birds with their beak anesthetized, in contrast, being deprived of the irregular magnetic input, seemed to turn to other cues right away.

These observations suggest that magnetic information is normally included in navigational decisions. Yet it is only one factor in the multi-factorial navigational ‘map’, which appears to be redundant to a certain degree. Pigeons released in Italy with their trigeminal nerve sectioned did not show navigational deficits (Gagliardo et al. 2008, 2009); the pigeons with the beak anesthetized in the above-mentioned study (R. Wiltschko et al. 2010b) did not show any effect at three of the four control sites, yet at the fourth site, they showed larger deviations from home, shorter vectors and longer vanishing intervals. Apparently, the significance of magnetic cues varies considerably between sites. But they are regularly consulted, as the obvious confusion of the untreated control birds within the anomaly suggests.

## Conclusion

Behavioral evidence indicates that there are magnetoreceptors in the beak of birds. These receptors include magnetite, as indicated by the pulse experiments, and they mediate their input to the brain by the ophthalmic nerve and the trigeminal system. They are not involved in the avian magnetic compass; instead, they seem to normally convey information on magnetic intensity. Their natural function appears to be to provide birds with magnetic information as one factor in the multi-factorial navigational ‘map’—not only homing pigeons within their home region, but also migrants when they return to their familiar breeding site or wintering area.

The exact position of these magnetite-based receptors is unclear. The effect of the local anesthetic seemed to speak in favor of the receptors described in the skin of the upper beak (e.g., Hanzlik et al. 2000; Fleissner et al. 2003; Falkenberg et al. 2010), yet the histological study by Treiber et al. (2012) calls the existence of these receptors in question, a finding that received considerable public attention. This may point to the single-domain receptors described in the nasal region (e.g., Beason and Nichols 1984, Beason and Brennan 1986; Williams and Wild 2001), but it appears highly unlikely that the externally applied anesthetic could have reached them. The observation that young chickens with the tip of their beak removed, as routinely done in the poultry industry, were impaired in locating a magnetic anomaly (Freire et al. 2011) also suggests a position of the receptors further in front of the beak.

Future histological studies will have to identify their true location and show details of their structure.

## Footnotes

1 The latter findings—normal preference of the migratory direction despite being treated with a pulse—make possible non-specific side-effects of the pulse treatment seem highly unlikely.

2 The observation that birds with the ophthalmic nerve or the upper beak anesthetized oriented in their normal migratory direction and the beneficial effect of local anesthesia of the beak in a magnetic anomaly (see below) speak against adverse effects on other sensory systems or non-specific side effects of these treatments.
